# Tumour auto-antibody screening: performance of protein microarrays using SEREX derived antigens

**DOI:** 10.1186/1471-2407-10-627

**Published:** 2010-11-16

**Authors:** René Stempfer, Parvez Syed, Klemens Vierlinger, Rudolf Pichler, Eckart Meese, Petra Leidinger, Nicole Ludwig, Albert Kriegner, Christa Nöhammer, Andreas Weinhäusel

**Affiliations:** 1 Molecular Medicine, Austrian Institute of Technology, Seibersdorf, Austria; 2 Department of Human Genetics, University of Saarland, Homburg, Germany

## Abstract

**Background:**

The simplicity and potential of minimal invasive testing using serum from patients make auto-antibody based biomarkers a very promising tool for use in diagnostics of cancer and auto-immune disease. Although several methods exist for elucidating candidate-protein markers, immobilizing these onto membranes and generating so called macroarrays is of limited use for marker validation. Especially when several hundred samples have to be analysed, microarrays could serve as a good alternative since processing macro membranes is cumbersome and reproducibility of results is moderate.

**Methods:**

Candidate markers identified by SEREX (serological identification of antigens by recombinant expression cloning) screenings of brain and lung tumour were used for macroarray and microarray production. For microarray production recombinant proteins were expressed in *E. coli *by autoinduction and purified His-tag (histidine-tagged) proteins were then used for the production of protein microarrays. Protein arrays were hybridized with the serum samples from brain and lung tumour patients.

**Result:**

Methods for the generation of microarrays were successfully established when using antigens derived from membrane-based selection. Signal patterns obtained by microarrays analysis of brain and lung tumour patients' sera were highly reproducible (R = 0.92-0.96). This provides the technical foundation for diagnostic applications on the basis of auto-antibody patterns. In this limited test set, the assay provided high reproducibility and a broad dynamic range to classify all brain and lung samples correctly.

**Conclusion:**

Protein microarray is an efficient means for auto-antibody-based detection when using SEREX-derived clones expressing antigenic proteins. Protein microarrays are preferred to macroarrays due to the easier handling and the high reproducibility of auto-antibody testing. Especially when using only a few microliters of patient samples protein microarrays are ideally suited for validation of auto-antibody signatures for diagnostic purposes.

## Background

The idea of early diagnosis of the onset of a disease via biomarkers has inspired several molecular biological approaches. In the past decade, since the unravelling of the human genome to a large extent, genomics technologies have been used to identify disease biomarkers. For cancerous diseases recently the most promising results were obtained by gene expression profiling. Excellent results have been achieved with these techniques in terms of improved patient stratification and increased potential of a clearer prognosis by a more detailed initial diagnosis. However, the true challenge is to develop techniques which are suitable for early diagnosis and prophylactic screening. These techniques should be minimally invasive, cost effective and ideally they indicate several diseases of the screened patient [1].

Proteomics techniques have shifted biomarker identification and validation research to the level of the main actual biological agents of health and disease, the proteins. Despite recent improvements in separation techniques based on HPLC (High-performance liquid chromatography) separation, mass spectrometry and 2D electrophoresis, so far the novel biomarker candidates and biomarker signatures which are ready for the use in clinical settings have proven to require detection steps similarly complex as in their discovery and thus complicate their widespread use in the screening of large populations [1].

Ideally the proteomics based techniques result in the identification of marker molecules that can be targeted in specialized assays relying on antibodies or aptamers [2-4]. The development of specific capturing agents for the candidate markers requires a costly production process and thorough validation. This ensures high avidity for the target while minimizing the risk of unspecific binding.

In the auto-antibody approach these issues are sophisticatedly avoided. The need to identify aberrant nucleic acid sequences, disease related biochemical compounds, disease affected cells or their debris is reduced by making use of a highly sensitive detection system closest to the patient, the human immune system. Mutated, modified and aberrantly expressed proteins evoke an immunological response leading to the production of auto-antibodies [5,6]. The auto-antibody based biomarkers could be used as serological tool for the early diagnosis and prognosis of cancer as auto-antibodies are specific to each kind of cancer [5,7,8]. Most of the auto-antibodies are immunological finger prints of pathological processes which are involved in the development of autoimmunity [5]. Such a molecular finger print of auto-antibodies which is produced against certain disease states can be called auto-antibody signature [7]. Assays for the detection of auto-antibodies at present are mainly ELISA (enzyme linked immunosorbent assay) and fluorescence immunoassays. However, protein microarrays have great potential to characterize auto-antibodies [9].

Strategies like the SEREX have been developed for the serological definition of immunogenic tumour antigens [10-13]. A similar approach has been used successfully for the identification of tumour endothelium associated antigen genes from human liver cancer vascular endothelial cells by generating a cDNA expression library. For the identification of auto-antibodies against pancreatic ductal adenocarcinoma-associated antigens which could be useful for early cancer diagnosis and therapy, a proteomics approach was followed up [14,15]. Proteins from pancreatic ductal adenocarcinoma cell lines were separated by 2D electrophoresis, and the serum IgG (immunoglobulin G) reactivity was tested by Western blot analysis. Spots specifically reacting with auto-antibodies from the sera of pancreatic ductal adenocarcinoma patients, which were analyzed by mass spectrometry, corresponded to metabolic enzymes or cytoskeletal proteins which proved to be specific targets of the humoral response to pancreatic ductal adenocarcinoma.

Over recent years most approaches have used so called macroarrays for autoantigen-profiling. These macroarrays are generated by spotting cDNA expression clones on membranes. Expression clones are grown on these membranes and recombinant proteins over-expressed upon induction are directly immobilized on the reactive membrane surfaces. Because entire colonies are lysed directly on the membranes and proteins of interest are immobilized in the background of the proteins of the expression-host bacteria, the targeted proteins are accessible for detection only after removal of the reactive anti-*E. coli*-Ig (immunoglobulin) from the analyte. This can be achieved either by masking of the anti-*E. coli *antibodies in human sera by addition of saturating concentrations of *E. coli *crude protein extracts and by blocking unwanted reactivity against *E. coli *by repeated incubation of membranes with the human serum. In this latter approach reactive Ig's from sera are captured by the macroarrays, sera are collected upon this primary incubation and after washing/stripping the membranes the sera are applied again onto the membranes obtaining then the signal from the Ig's specific for the reactive over-expressed antigens. Handling membranes and processing sera is cumbersome, and sensitivity and reproducibility of these macroarrays are limiting. Signals derived from membranes are not dynamic. In analogy to western blotting different strategies exist to enhance sensitivities and to extend the dynamic range of membrane-based measures, but are rather limiting compared to the 16 bit (0-2^16^) dynamic range of standard microarrays.

In this study we optimized methods for generation of protein microarrays and provided an optimized protocol for generation of biomarker profiles with high reproducibility using 10 μL amounts of patient serum samples. The provided data do also confirm that clones derived from SEREX membrane screens can be successfully transferred onto microarray slides retaining reactivity and gaining dynamic signal measures suitable for class-comparison to elucidate and validate protein-biomarkers.

## Methods

### Candidate marker screening

The candidate markers were identified by previous SEREX screenings of brain and lung cancer, and screening macroarrays of a fetal brain cDNA expression library. Potential tumour associated antigens derived from SEREX screens were isolated and sub cloned in the expression vector pQE30NST for production of His-tag (histidine-tagged) fusion proteins [16-20]. Marker candidate screening involved testing of serum from lung tumour patients and from brain tumour patients as well as control sera under the patients' informed consent. The local ethics committee (Ärztekammer des Saarlandes, Kenn-Nr. 213/08) approved the study and the research was carried out in compliance with the Helsinki Declaration.

### Protein Microarray production

*E. coli *culturing and induction was performed in 96well format with slight modifications [20]. Recombinant protein expression was induced either by IPTG (Isopropyl β-D-1-thiogalactopyranoside) or by cultivation of bacterial clones in autoinduction medium (1 mL) [21]. Upon cultivation His-tagged recombinant proteins were purified using Ni-NTA (nickel immobilized onto agarose resin via nitrilo triacetic acid) agarose and chosen elution conditions were adopted warranting protein-binding onto ARChip Epoxy coated slides [22]. Elution of His-tag protein was done using 500 mM imidazole.

Purified proteins were electrophoresed and analyzed using standard procedures. Protein eluates from Ni-metal-chelate purification were controlled for specificity via a His-Tag antibody ELISA. Protein antigens were printed in triplicates on ARChip Epoxy glass slides. Crude clarified protein extracts of the *E. coli *host was used for positive control spots, plain buffer spots were used as negative controls. The detailed protocols are available from the supplement (see additional file 1: detailed information on methodology).

### Assay protocols

Reactive groups on the slide surface were blocked for 2 h in PBST (Phosphate buffered saline with 0.1% Tween 20) blocking buffer with 5% non-fat milk powder. Slides were washed 2 times 5 min in PBST wash buffer, rinsed with distilled water and blown dry with filtered air. Arrays were incubated for 1 h with patients' sera and control sera diluted 1:10 in blocking buffer. Upon washing twice for 5 min in wash buffer, slides were rinsed with distilled water and blown dry with filtered air. Arrays were incubated for 1 h with goat anti human IgG detection antibody fluorescently labelled with *Alexa647 *dye (Invitrogen, Lofer, Austria), diluted 1:500 in blocking buffer. Following the final washing steps of twice 5 min washing in wash buffer, arrays were rinsed with distilled water and dry-blown. Array images were captured using an Axon Genepix 4000A microarray scanner (Molecular Devices, Union City, CA).

### Data analysis

Fluorescence intensities - medians after subtraction of the local background - were calculated from the scanned array images with the Genepix software (Molecular Devices, Union City, CA). Statistical data analysis was performed using R version 2.6.2, BRB-ArrayTools Version: 3.6.0 - Stable Release, limma software package and nearest shrunken centroid algorithm [19,23,24]. The nearest shrunken centroid algorithm is used to find out the clusters in the samples using hierarchical clustering methods on expression arrays [25].

## Results

### Methods optimization

The bacterial wet biomass (30 mg/mL culture) obtained by autoinduction was twice when compared to that of obtained from IPTG cultures. Using Ni-NTA-metal chelate purification the amount of purified protein from 1 mL of bacterial culture (autoinduced) was 7-70 ng at an average concentration of about 0.2-0.25 mg in 75 μL of elution-buffer. Protein yields were similar with both methods which points towards similar effectiveness in protein expression. The expression rate of recombinant proteins was 40% for both IPTG and autoinduction as determined by Penta-His antibody ELISA. Although there were some minor differences between different batches of 1 mL bacterial cultures grown in 96well plates and distinct runs of protein-purification it becomes clear that both induction methods of recombinant protein expression were equally successful (see additional file 1: detailed information on methodology). When several repeated experiments did not show a great difference between both induction strategies, we continued with the autoinduction method because of easier technical handling.

It was found out that the amount of His-tag protein yielded upon elution using 250 and 500 mM imidazole to be more or less the same. Eventually, we used 500 mM imidazole for elution of all proteins which were used for microarray printing.

Optimizations for processing the protein-arrays covered the 1) blocking-reagent, 2) serum-incubation time, and 3) detection of serum-auto-antibodies using anti-humanIg-Alexa647 conjugate. Addition of 5% non-fat- dry milk into PBST was efficient when blocking slides for 30 min at room temperature. Prolonged blocking did not significantly increase signal to noise ratios. Although bovine serum albumin has been described for blocking, milk powder is an efficient and an inexpensive alternative. Omission of the milk powder, however led to strong unspecific binding of serum-proteins to the microarray and thereby to high background signals.

We tested serum-incubation time with respect to signal intensities of microarray spots. Using a 1:10 dilution of sera from healthy controls in PBS (Phosphate buffered saline) signal intensities reached a plateau after 2 h incubation at room temperature. Signal intensities upon 4 h incubation were comparable to intensities after 2 h incubation. Therefore, a 2 h serum-incubation step was used for all further tests and found to give sufficiently high signals to identify clear and distinct auto-antibody patterns from controls as well as from patient's samples. (See additional file 2: figure displaying the detailed layout of the antigen-microarray). Negative control experiments conducted without serum excluded unspecific binding of the anti-human Ig-Alexa647 detection antibody and indicate that the detection step is specific to human auto-antibodies. Thus, spot signals are derived from specific serum antibody-binding with the antigens presented on the chip. No direct correlation of the amounts of spotted protein with the yielded signal strengths could be detected. Hence, the yielded signals are due to the presence of antibodies specific to the target proteins and not to unspecific binding which would clearly correlate with protein mass. Positive control spots of *E. coli *crude protein extracts showed high signals indicating the presence of high levels of antibodies against *E. coli *proteins in the sera of all donors and patients, whereas buffer spots serve as controls were clearly negative. Thus, false positive-signals derived from carry-over of reactive proteins from printing spots with the same set of pins during microarray fabrication can be excluded. (See additional file 2: figure displaying the detailed layout of the antigen-microarray). Here, we want to mention that recombinant proteins derived from single step Ni-His(6)-affinity protein purification are not pure and will contain several percentages of *E. coli *proteins, which could be problematic when covering specific signals. This, however, might be especially true for primary-screens to identify specific antigens from clone libraries. In our setting all clones used for antigen-purification and microarray fabrication were selected via several pre-screens within the SEREX procedure (see methods and additional file 1: detailed information on methodology). Reactivity of the spotted proteins was not covered by serum-reactivity against remaining *E. coli *proteins, therefore, the microarray enabled a specific and clear differentiation between sera derived from lung cancer and brain cancer patients.

**Figure 1:**
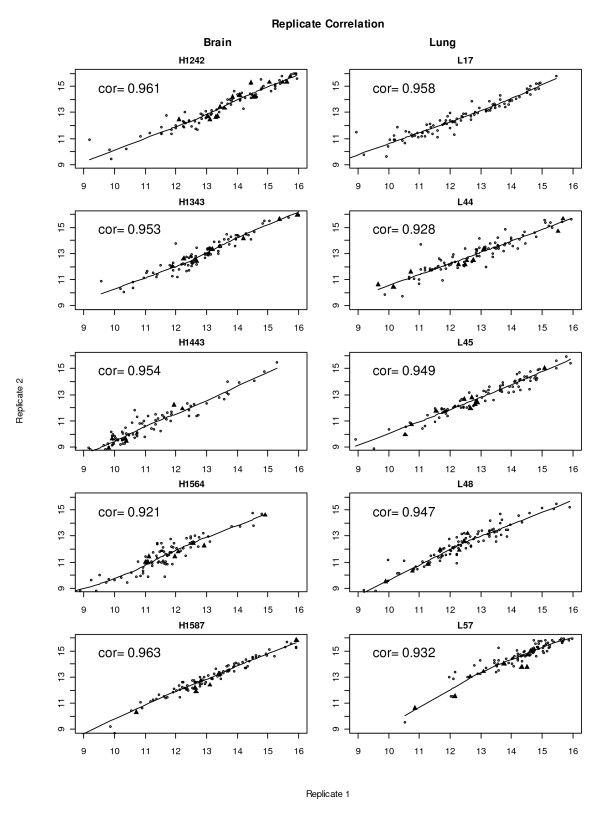
**Pair wise correlation of repeated protein microarray analyses**. Pair wise correlation of repeated analyses of serum samples. Log2 transformed unnormalised intensities, with a threshold set to 512 intensity-units (derived from Genepix .gpr files) were used for analyses. Correlation coefficients are given in the paired scatterplots and were above 0.92 upon repetitive analyses. The "filled triangles" represent reactive clones from each individual serum found within membrane-based macroarray testing. Data from repetitive analyses (replicate-1 on x-axes; replicate-2 on y-axes) microarray analyses using serum from brain (left) and lung (right) tumour patients (identifiers of different patient sera on top of each scatter plot) are plotted.

Serum samples have been initially tested during the SEREX-membrane screen. Some subsets of clones which have been tested positive with several patient sera were used for evaluation of protein microarrays. On the chip, binding of auto-antibodies to the candidate marker proteins was observed as demonstrated in the initial SEREX screens (Figure 1). Binding events detected in addition to the marker candidates that were expected from macro-membrane screens (in analogy to SEREX screens) indicate greater detection limit/signal intensity of the microarray when compared to the membrane method. This might be due to the smaller reaction surfaces and better distribution of the serum-sample over the array (Figure 1). Moreover, this greater detection limit/signal intensity is achieved with a few microliters of analyte i.e., 1:10 diluted sera (about 75 μL is sufficient for wetting the entire area of a standard slide).

### Performance of microarray based serum-auto-antibody testing

**Figure 2:**
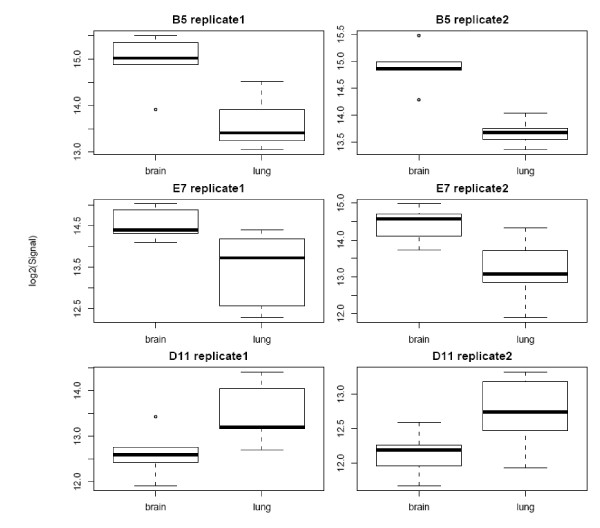
**Replicate measurements of the top three differentially reactive clones**. Performance of the top three differentially reactive clones (B5, E7 and D11 with p-values less than 0.002, 0.01, 0.05, respectively) in replicate experiments. The normalized signal intensity values of these reactive clones across the replicate measurements (replicate-1 and -2) distinguishes between brain (n = 5) and lung (n = 5) cancer serum samples.

**Figure 3:**
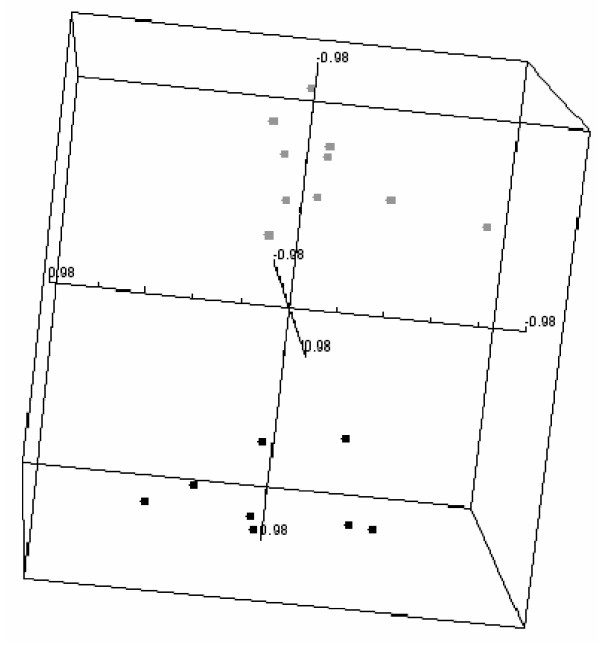
**Multidimensional scaling of protein microarray data of brain and lung tumour patients' sera**. Multidimensional scaling using centered correlation of significant antigens derived from class comparison (using 10 most significant antigens). Microarray data as depicted in scatterplots (figure 1) of duplicate analysis of brain (black) and lung (gray) cancer patients' serum samples were used for class comparison.

Detection using anti-human Ig-Alexa647 conjugate upon application of patients' sera to the blocked arrays yielded clearly visible binding patterns that already at an optical level displayed almost identical patterns. Also the control sera yielded specific patterns, yet different to the ones of patient's sera. Pair-wise correlation plots of repetitive serum-testing on different slides confirmed the high reproducibility of the signal patterns and results in correlation coefficients ranging from 0.92 to 0.96 of (Figure 1). Statistical data analyses of brain and lung cancer serum-microarray data was performed in analogy to gene-expression microarray data analysis using the limma software package. Figure 2 shows the normalised signal intensities of the three most differentially reactive clones between brain and lung. It shows that across replicate measurements the assay is capable of distinguishing these two biological classes. For these genes, both technical variances and within-group variances are small, compared to the between-group variances. Therefore it is not surprising, that when attempting to build a classification rule using the nearest shrunken centroid algorithm on replicate-1 and testing this rule on replicate-2, all samples are classified correctly. In the reverse case (building the rule on replicate-2 and testing it on replicate-1), one sample is misclassified. The good separation between the two classes is also visualised in Figure 3.

## Discussion

Auto-antibodies are very potent biomarkers which would be useful for minimal invasive testing for early diagnosis of autoimmune and cancerous disease. Beside SEREX-based screening using immobilized expression clones on membranes, macroarrays with several thousand expression-clones derived from human cDNA libraries are suitable platforms for screening for determining reactive clones over-expressing proteins which are biomarker-candidates [19]. However, the membrane based clones are not a versatile tool for validation of those candidate makers. Drawbacks of membrane based screening are low reproducibility, low dynamic range of signal intensities, and difficulties in handling membranes. In addition to these technically problems, several hundred microliters of patient serum for processing the membranes or macroarrays are required. Because sample size of clinically well documented samples is always limited, miniaturization of assays using microarrays would be a great option to save samples (about 75 μL of sample is sufficient to cover an entire 1 × 2 inch standard slide). As known from the performance of DNA-microarrays, obtaining high reproducibility, high dynamic range of intensity-measures (usually in the range of 4-6 orders of magnitudes; derived form 16 or 20 bit microarray scanners) and easy handling microarrays, protein microarrays would be a potential alternative for validation of disease specific serum-auto-antibody profiles.

Here we set out to generate protein microarrays and evaluate their performance with respect to technical aspects like reproducibility and suitability using patient serum samples already used for candidate biomarker screening. Therefore we set up techniques and optimized with respect to 1) recombinant protein expression from candidate clones, 2) protein-purification in a 96well standard plate format and microarray printing, and 3) finding best conditions of serum-testing on antigen-microarrays (see methods and additional file 1: a detailed information on methodology). We have found during optimization (data not shown) of protein microarray production that proteins concentrations of up to 0.5 mg/mL are well suited for spotting using a contact spotter. At that protein-/antigen-concentrations microarrays perform well (with respect to signal intensities and spot morphology) and at that concentration clogging of microarrayer-pins is also avoided.

Upon protein-purification using His-tag/Ni-affinity we measured protein-concentrations and determined specific recombinant proteins using a His-tag-ELISA. Although we have not compared microarrays generated from different batches of protein-purification the ratio of His-tag-ELISA signals and protein-concentration would be a practicable measure of "purity" which should be taken into consideration when using different protein-batches for microarray generation. Although the "different slide batch effect" is known also from DNA-chips, this would be clearly more critical using proteins derived from different batches of clone-cultivation, expression and purification. Therefore, while using (protein) microarrays for screening purposes defining biomarkers would be done best when using the same batch of microarrays avoiding these effects. When not avoidable that must be considered by proper experimental planning. We found out that the membrane-blot derived classifiers (which enabled distinction of brain and lung tumour serum antibody profiles) did perform well also on the microarray-derived data set, confirming the reliability of the reactive markers. This is true even when data were derived from two entirely distinct methodologies. Membrane blots are generated by fixation of proteins upon growing *E. coli *clones on membranes and microarrays are spotted using proteins from distinct 1 ml culturing of clones. Optimized conditions for obtaining maximum signal intensities on microarrays were achieved with 1:10 serum dilutions after 2 h incubation at room temperature. Thus arrays covering an entire standard slide can be processed with only 10 μL of serum. This would enable paralleled detection of about 20000 different spots, a spot density usually achieved with standard microarray printing techniques. We could also show the high reproducibility of protein microarray-data. Correlation coefficients of repeated analyses using patient sera were in the range of 0.919-0.971 (median 0.957). While the differentially reactive clones identified in this study need more independent testing to prove their usefulness as clinical markers, we have shown that the assay is capable of detecting differences between biological groups which are stable and reproducible and are therefore suitable for class comparison and class prediction. Thus, this kind of microarrays has several advantages over macroarrays and microarray based testing of patient samples is the method of choice for highly paralleled auto-antibody testing. Especially when many different samples have to be processed for validation of biomarker candidates, handling many microarrays is much easier and also for screening approaches microarrays are best suited and will replace membrane-based macroarray screens. Biostatistical analysis of high-dimensional data derived from microarray-feature intensities is also well established and can be used in the analysis of auto-antibody data.

## Conclusion

In conclusion, herewith, we successfully demonstrate the feasibility for auto-antibody identification technology by means of recombinant protein expression and arraying the proteins on microarray solid supports. Because panels of auto-antigens rather than individual antigens enhance the likelihood of detecting cancer antigens with diagnostic potential [26-28], highly paralleled detection of auto-antibody signatures yielded from this platform will be aiding disease diagnosis and improve patient stratification [19,29].

## Competing interests

The authors declare that they have no competing interests.

## Authors' contributions

RS and PS contributed equally in doing the experiments and writing the manuscript. RP printed the microarrays. EM, PL and NL provided the SEREX clones. AK, KV, CN and AW did the statistical data analysis. AW is the corresponding author. All the authors have read and approved the final manuscript.

## Pre-publication history

The pre-publication history for this paper can be accessed here:

http://www.biomedcentral.com/1471-2407/10/627/prepub

## Supplementary Material

Additional file 1 **Supplemental information**. Detailed information on methodology Click here for file

Additional file 2 **Microarray layout**. Figure displaying the detailed layout of the antigen-microarray Click here for file
