# Supplementary material for: Tumour auto-antibody screening: performance of protein microarrays using SEREX derived antigens
Source: BMC Cancer. 2010 Nov 16;10:627. doi: 10.1186/1471-2407-10-627 (PMC2995456; doi:10.1186/1471-2407-10-627)
Supplement: Additional file 1 — Supplemental information. Detailed information on methodology [file 1471-2407-10-627-S1.PDF]

Supplemental information

E. coli culturing and induction:

E. coli culturing techniques were adapted and modified after [1,2]. Deep-96well micro titre plates were filled with 1200µL 2xYT medium (per L: Bactotryptone 16g, yeast extract 10g, NaCl 5g) supplemented with 2% glucose, 100µg/mL ampicillin, and 15µg/mL kanamycin. Plates were inoculated with 3µL from master plates of 96 *E. coli* cultures (using vector constructs of the SEREX library) each, sealed with gas permeable film and incubated overnight at 37°C while shaking at 1000 rpm. For the glycerol daughter plates, in each well 20µL glycerol were combined with 50µL of the overnight cultures and stored at -80°C.
Deep-96well microtiter plates were filled with 100µl 2xYT medium with the aforementioned supplements. Media were inoculated with 3µL of the glycerol stocks. Plates were sealed with gas permeable film and shaken overnight at 37°C. In the morning, 900µL SB (Sabouraud broth) medium pre-warmed to 37°C were added to each well.

One litre of SB medium consisted of: bactotryptone 12g, yeast exctract 24g, glycerol 4mL, potassium phosphate buffer 50mL (consisting of 2.4g KH_2PO_4 and 12.15g K_2HPO_4 in 50mL) were added per litre medium; upon autoclaving the medium was supplemented with stock solutions obtaining final concentrations of 100µg/mL ampicillin, 15µg/mL kanamycin, and 20µg/mL thiamine.)

Plates were sealed with gas permeable film and shaken at 37°C at 1000rpm until an OD_{600} of 0.5-0.8 was reached for the IPTG (isopropyl-β-D-thiogalactopyranosid) induction. Then, 100µL 11mM IPTG in distilled water was added and cultures were shaken for 3h at 37°C at 1000rpm. *E. coli* cells were pelleted for 10min at 3500rpm, and the supernatants were

discarded. Pellets were washed in PBS (phosphate buffered saline, pH 7.0) and the washed pellets were frozen at -80°C pending protein extraction.

Alternatively, for the autoinduction protocol, the SB medium was additionally supplemented with 0.5% glycerol, 0.05% glucose, and 0.2% lactose. Upon addition of SB medium, plates were shaken overnight at 30°C at 1000rpm and then pelleted.

Comment on induction-strategies for protein-expression:

In order to determine the ideal time-point for induction of the *E. coli* culture with IPTG, the OD had to be controlled tightly with a set of test clones over a course of 0.5 to 3h. Since these test clones may not ideally represent all the clones, and measuring the OD of all clones simultaneously is not feasible, the optimum time-point of induction may not be met by several clones. The autoinduction principle of William F. Studier [3] has been modified and adapted for the use in complex media to overcome this issue of having to choose the ideal time for the addition of an inducer. The minute amounts of glucose in the medium prevent untimely induction by the present lactose until high cell densities are reached. Then, when all of the glucose is metabolized, and with no other nutrients limiting, the less attractive nutrient lactose is taken up by the cells and then causes induction. With IPTG induction, protein concentrations after the Ni-NTA affinity purification on average reached 0.26mg/mL while with the auto-induction concentrations of 0.21mg/mL were achieved. These similar yields in protein concentration point towards similar effectiveness in protein expression. Considering the rate of clones expressing His-tagged protein of 41% of the IPTG-induced clones as determined by the Penta-His antibody detection on chip and the expression rate of 40% of the auto-induced clones as determined by the His-tag ELISA, it becomes clear that both methods of induction of recombinant protein expression were equally successful.

Protein extraction:

Bacterial pellets were thawed and resuspended in 100µL lysis buffer (50mM Tris-HCl, pH 8.0, 0.3M NaCl, 0.1mM EDTA, 0.02 % NaN_3). Lysozyme was added to a final concentration of 50µg/well in a 1% Brij58 solution. Pellets were resuspended and incubated on ice for 30min to yield the whole cellular extract. To each well 25µL of benzonase mixture were added containing 0.3µL 1M $MgCl_2$, 0.1µL benzonase grade 11, and 24.6µL 50mM Tris-HCl. Lysates were mixed, incubated at RT (room temperature) for 30min and centrifuged for 30min at 6200rpm at 4°C for removal of cell debris. The supernatant was transferred to a small-pore filter plate (MSDVN 6510, Millipore GmbH, Vienna, Austria) to remove any remaining particles. Filter plates were placed on top of capture plates and supernatants were filtered by centrifugation at 1000g obtaining the clarified protein extracts.

Protein purification:

The filtered protein extracts were transferred to small-pore filter plates (MSHVN 4510, Millipore GmbH, Vienna, Austria) placed on top of a capture plate. Then, 15µL of 0.1M imidazole were added to each well to reach an end concentration of 10 mM. Upon addition of 25µL of 20% Ni-NTA agarose (Qiagen, Hilden, Germany) equilibrated in 50mM Tris-HCl, pH 8.0, samples were shaken for 30min at RT. Liquid was removed by centrifugation of the filter plates at 1000 g followed by three washing steps with sodium phosphate wash buffer (50mM monobasic and 50mM dibasic sodium phosphate mixed together to produce a solution with pH 8.0), 0.3M NaCl, and 20mM imidazole. Upon the last wash protein was eluted with 50µL of the elution buffer (sodium phosphate buffer as abovementioned, pH 8.0 containing 250mM or 500mM imidazole, 0.01% SDS and 0.01% NaN_3).

Electrophoresis:

Purified protein samples of 5µL were resolved on NuPAGE Novex 4-12% Bis-Tris gels (Invitrogen) in the MES SDS running buffer system (Invitrogen, Lofer, Austria). PageRuler prestained protein ladder (Fermentas GmbH, St. Leon-Roth, Germany) was used as a standard. Gels were stained first with the 6xHis Protein Tag staining kit (Pierce Biotechnology, Rockford, IL) according to manufacturer's instructions, and then according to the conventional protocol of the PageBlue protein staining solution (Fermentas GmbH, St. Leon-Rot, Germany).

Determination of protein-concentrations in microtiter plate:

The DC Protein Assay from kit II (Bio-Rad, Hercules, CA, USA) was adapted for the use in microtiter plates. BSA (bovine serum albumin) standards were prepared in elution buffer. Pipetting volumes of the protein assay were scaled according the manufacturer's instruction for using 5µL of sample-volumes for the measurements. Absorbance at 630nm was read on an ELx800 automated microplate reader (Bio-Tek Instruments, Inc., Bad Friedrichshall, Germany) and protein-concentrations calculated based on the BSA standard measures.

ELISA-measurement of His-tagged proteins:

For the relative quantification of His-tagged proteins an ELISA assay was established employing the Penta-His HRP (horseradish-peroxidase) conjugate detection antibody. Samples were diluted in immobilization buffer (50mM sodium carbonate buffer; 100mM sodium carbonate and 100mM sodium bicarbonate mixed together to produce a solution with

pH 9.6) to a total protein content of 10µg/mL. 100µL of the samples were transferred to high-binding Nunc MaxiSorp 96well plates (Nunc GmbH & Co. KG, Thermo Fisher Scientific, Wiesbaden, Germany) and incubated overnight at 4°C. The solution was removed and 200µL blocking solution (50mM Tris, pH 8.0, 0.14M NaCl, 1% BSA) was added for an incubation of 30min at RT. The solution was removed and 100µL HRP-conjugated antibody were added to each well in the required dilution of 1:100,000 in conjugate diluent (50mM Tris, pH 8.0, 0.14M NaCl, 1% BSA, 0.05% Tween 20) for an incubation of 60min at RT. Plates were washed 5 times with ELISA wash buffer (50mM Tris, pH 8.0, 0.14M NaCl, 0.05% Tween20). Equal volumes of the two-substrate reagents TMB peroxidase substrate (Kierkegaard & Perry Laboratories, Gaithersburg, MD) and of solution B (Kierkegaard & Perry Laboratories, Gaithersburg, MD) were mixed and 100µL of this mixture were added to each well. Plates were incubated at RT for 5-30min until colour development and stopped by the addition of 100µL 2M H_2SO_4 to each well, added in the same order as the enzyme substrate. Plates were read at 450nm on the ELx800 automated microplate reader (Bio-Tek Instruments, Inc., Winooski, VT).

Protein arraying:

Protein antigens were spotted using an Omnigrid arrayer (GeneMachines, San Carlos, CA) with SMP 3 pins (TeleChem International Inc., Sunnyvale, CA) under adjusted air humidity of between 55% and 60%. Spots were printed in triplicates on ARChip Epoxy [4] glass slides. Crude clarified protein extract of the *E.coli* host was used for positive control spots, plain buffer spots were used as negative controls.

Quality control of the spotting process:

The recombinant protein expression, the spotting process and the immobilization of protein on the chip surface were controlled by binding of an anti-Penta-His Alexa Fluor 647 conjugate antibody recognizing groups of five histidines in the Hexa-His-tag. To the blocked slides, detection antibody was added in a 1:50,000 dilution in blocking buffer. Slides were washed twice in wash buffer and blown dry with filtered air.

Reference list

1. Bussow K, Cahill D, Nietfeld W, Bancroft D, Scherzinger E, Lehrach H *et al.*: **A method for global protein expression and antibody screening on high-density filters of an arrayed cDNA library.** *Nucleic Acids Res* 1998, **26:** 5007-5008.

2. Büssow K, Nordhoff E, Lubbert C, Lehrach H, Walter G: **A Human cDNA Library for High-Throughput Protein Expression Screening.** *Genomics* 2000, **65:** 1-8.

3. Studier FW: **Protein production by auto-induction in high density shaking cultures.** *Protein Expr Purif* 2005, **41:** 207-234.

4. Preininger C, Bodrossy L, Sauer U, Pichler R, Weilharter A: **ARChip epoxy and ARChip UV for covalent on-chip immobilization of pmoA gene-specific oligonucleotides.** *Anal Biochem* 2004, **330:** 29-36.
