# Supplementary material for: Tumour auto-antibody screening: performance of protein microarrays using SEREX derived antigens
Source: BMC Cancer. 2010 Nov 16;10:627. doi: 10.1186/1471-2407-10-627 (PMC2995456; doi:10.1186/1471-2407-10-627)
Supplement: Additional file 2 — Microarray layout. Figure displaying the detailed layout of the antigen-microarray [file 1471-2407-10-627-S2.PDF]

Microarray layout

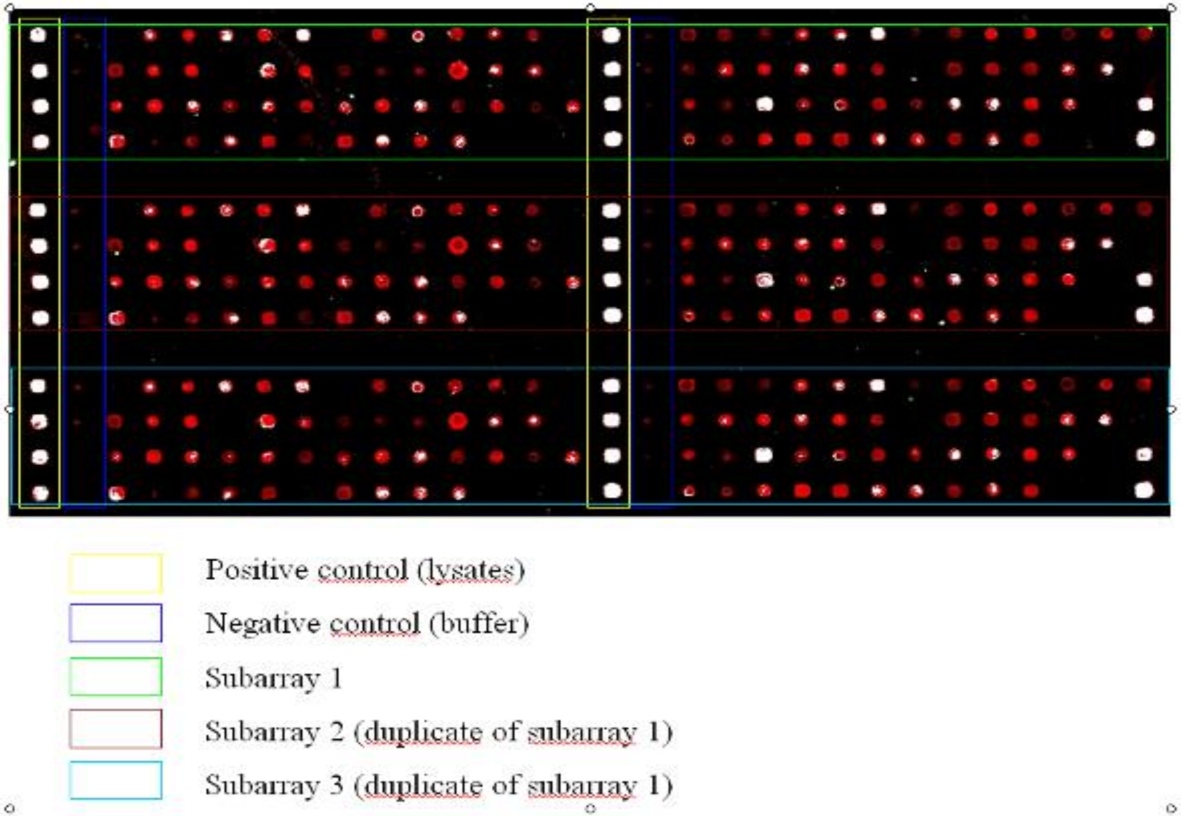

Layout of the antigen-microarray. Microarray consisting of 3 identical sub arrays (4 rows x 30 columns) of each 96 proteins, positive and negative spotting-controls. A mixture of crude cell lysates was used as positive control spots (bright spots – entire columns 1 and 16, and in row 3 and 4 –in column 30), buffer spots were used as negative control spots (blank columns 2, 17 and row 1-column 3, row 4-columns 13-15, 28, 29; see also colour coded boxed areas as indicated in the bottom of the figure).
